# Assessment of the Effect of School Feeding on Dropout Rate in Kolfe Keranio Public Primary School, Addis Ababa, Ethiopia: A Quasi‐Experimental Study

**DOI:** 10.1155/sci5/9862553

**Published:** 2026-02-15

**Authors:** Mequanente Dagnaw, Eden Hailu, Suleyman Mohammed Arage

**Affiliations:** ^1^ Department of Epidemiology and Biostatistics, Institute of Public Health, University of Gondar, Gondar, Ethiopia, uog.edu.et; ^2^ Department of Medical Biotechnology, Institute of Biotechnology, University of Gondar, Gondar, Ethiopia, uog.edu.et; ^3^ Department of Nurse, GT Technology College, Gondar, Ethiopia; ^4^ Department of Project Management, Select College, Addis Ababa, Ethiopia; ^5^ Department of Public Health, College of Medicine and Health Science, Werabe University, Worabe, Ethiopia; ^6^ Department of Health System and Policy, Institute of Public Health, College of Medicine and Health Science, University of Gondar, Gondar, Ethiopia, uog.edu.et

**Keywords:** dropout rate, Kolfe Keranio subcity, school feeding program

## Abstract

**Introduction:**

School feeding programs aim to reduce children’s immediate hunger and improve health and education outcomes. The study will apply trend analysis to investigate the changes in dropout rate before and after implementing the school feeding program. However, there is limited data on the effect of school feeding programs on dropout rates in Addis Ababa, especially in the study area.

**Objective:**

The objective of the study was to assess the effect of the school feeding program on the dropout rate in Kolfe Keranio subcity, Addis Ababa, Ethiopia, 2024**.**

**Methods:**

Quantitative research approaches were employed to investigate the problem. Twenty‐three primary schools, from grade one to grade 8, were included in the research. A school roster document was a source of quantitative data. A statistical test, a one‐group pretest–post‐test design, was used to measure the differences in dropout rate before and after the program’s implementation.

**Result:**

The results of the retrospective study revealed that each year’s female students had a higher dropout rate than male students before as well as after the implementation of the school feeding program. Concerning the trend from 2009 to 2015, the dropout rate decreased from 3.18% to 0.91% from 2009 to 2012 (before school feeding implementation), and an up‐and‐down trend was observed from 2012 to 2015 (after school feeding program implementation). However, the magnitude of the dropout rate was lower from 2012 to 2015, which indicates that the after‐school feeding program had started. The paired sample *T*‐test also identifies that the decreased trend in dropout was really due to the school feeding program implementation.

**Conclusion:**

Based on the findings, the following recommendations were forwarded for Kolfe Keranio subcity for each woreda educational office and each public primary school needed to make a proactive effort to decrease the dropout rate by bringing out‐of‐school children into schools by advocating the benefits of the SFSIS program, and SFSIS implementers, with the help of educational planners, should set up more effective monitoring and evaluation mechanisms to provide all the SFSIS program components.

## 1. Introduction

### 1.1. Background

School feeding programs (SFPs) provide meals or snacks to children during the school day and are widely recognized as effective social protection interventions that link education, nutrition, and food security [1]. By reducing short‐term hunger and nutritional deficiencies, SFPs enhance concentration, learning readiness, and motivation, thereby supporting improved school enrollment, attendance, retention, and reduced dropout rates [2]. Globally, SFPs are among the most extensively implemented education‐related interventions, benefiting hundreds of millions of children each year, particularly in low‐ and middle‐income countries [3]. Their objectives include alleviating classroom hunger, improving nutritional status, increasing school participation, promoting gender equity, and strengthening long‐term human capital development [4]. Systematic reviews consistently show that SFPs are associated with increased attendance and modest but significant reductions in school dropout, especially among children from food‐insecure households [2].

In Sub‐Saharan Africa, persistent poverty and food insecurity have elevated the policy importance of SFPs, leading many countries to integrate them into national education and social protection strategies [5]. Regional evidence indicates that SFPs improve attendance and sustain enrollment, particularly among vulnerable groups such as girls and children from disadvantaged households [1]. In Ethiopia, despite substantial gains in access to primary education, dropout remains a challenge, driven by household poverty, food insecurity, and limited social support [6]. In response, the government has incorporated school feeding into national education policies [6]. In Addis Ababa, SFPs have been introduced in selected public primary schools, including those in Kolfe Keranio subcity, where socioeconomic inequality and food insecurity persist. However, school‐level evidence on the impact of SFPs on dropout in urban settings remains limited, underscoring the need for context‐specific evaluation.

### 1.2. Statement of the Problem

School dropout remains a major barrier to achieving universal primary education and equitable learning outcomes worldwide [7]. Despite substantial global investments aimed at expanding access to schooling, millions of children fail to complete the primary education cycle, indicating that access alone is insufficient to ensure sustained participation [8]. Children from socioeconomically disadvantaged households are disproportionately affected, as poverty, food insecurity, and unmet basic needs are strongly associated with irregular attendance and early school withdrawal [9]. When children are compelled to prioritize survival needs, household responsibilities, or income‐generating activities over schooling, educational participation becomes fragile, undermining human capital development and perpetuating intergenerational cycles of poverty and social exclusion [10].

Globally, household financial hardship and child malnutrition are among the most significant contributors to school dropout [11]. Hunger negatively affects concentration, cognitive functioning, and academic performance, increasing absenteeism, grade repetition, and the likelihood of school exit [12]. For children from food‐insecure households, school attendance alone does not guarantee effective learning when daily hunger disrupts engagement and persistence [13]. The consequences of school dropout extend beyond the individual, resulting in reduced lifetime earnings, higher unemployment, poorer health outcomes, and increased exposure to social risks, with broader implications for national productivity, economic growth, and social stability [14].

The burden of school dropout is particularly severe in Sub‐Saharan Africa due to persistent socioeconomic challenges, including poverty, food insecurity, child labor, illness, and limited access to social protection. Rapid population growth, unemployment, and rising living costs especially in urban areas further intensify household vulnerability. Regional studies consistently identify hunger and low household income as key determinants of poor attendance, grade repetition, and premature school withdrawal. However, most of this evidence is derived from rural‐ or national‐level analyses, with limited attention to urban contexts where poverty is often less visible but equally severe [15].

In Ethiopia, although access to primary education has expanded substantially, school dropout remains a major concern. National education reports indicate that a considerable proportion of students enrolled in public primary schools do not complete the primary cycle. Household poverty, food insecurity, low parental education, child labor, poor academic performance, and nutrition‐ and health‐related problems continue to undermine educational efficiency and hinder the achievement of national education goals [16].

In Addis Ababa, school dropout persists despite relatively better educational infrastructure, suggesting that economic vulnerability and household food insecurity remain key determinants of school participation. City‐level data show notable fluctuations in dropout rates over time. For instance, the overall primary school dropout rate declined to 0.57% in 2015 E.C. (2022/23 G.C) but was considerably higher in earlier years, reaching 2.55% in 2013 E.C. (2020/21 G.C) and 2.02% in 2011 E.C. (2018/19 G.C). Dropout rates also vary by educational cycle, with higher rates observed in lower grades. Although these percentages appear small, they represent substantial numbers of disadvantaged children exiting school prematurely within a large urban education system [17].

To address school dropout, interventions such as school fee abolition, social protection schemes, and SFPs have been implemented globally and nationally. School feeding has gained particular attention as a multisectoral strategy intended to reduce hunger and improve school participation and retention. In Ethiopia, SFPs have expanded in selected urban public primary schools, including Addis Ababa. However, existing studies largely focus on nutritional status or attendance outcomes, with limited emphasis on dropout as a primary outcome. Moreover, little is known about how variations in program implementation, such as coverage, consistency, and targeting across schools and subcities, influence dropout rates in urban settings. School‐level evidence remains scarce, particularly in low‐income urban areas. This study, therefore, seeks to address this gap by generating school‐level evidence on the impact of SFPs on dropout rates in public primary schools in Kolfe Keranio subcity, Addis Ababa, thereby informing policy decisions and program design aimed at improving student retention.

## 2. Methods

### 2.1. Study Setting and Period

This research was carried out in Addis Ababa, the capital city of Ethiopia [18], from January 10 to February 10, 2024. It was the country’s largest city and plays an important political, economic, and symbolic role in Ethiopia [18]. The city was divided into 11 subcities and 116 districts, and according to the 2007 population census, there were a total of 3,384,569 people, and the population will exceed 5 million in 2036 [18]. There were 349,696 primary school pupils in grades 1 to 8 [19], and the SFPs had now been implemented in all 264 public primary schools located in all subcities, with 638,857 students benefiting from the SFP.

### 2.2. Study Design

This study employed a quantitative, quasi‐experimental research design to assess the effect of the SFP on school dropout rates at Kolfe Keranio Public Primary School. Specifically, a one‐group pretest–post‐test design was used, in which outcome measures were collected from the same school population before and after the implementation of the SFP. This design was selected because the SFP had already been implemented as a public policy intervention, making randomization or the establishment of a parallel control group infeasible for ethical and administrative reasons.

### 2.3. Population

#### 2.3.1. Source Population

The source population consisted of all students enrolled in public primary schools in Kolfe Keranio subcity, Addis Ababa, Ethiopia, during the 2024/25 academic year.

#### 2.3.2. Study Population

The study population included students enrolled in selected public primary schools in Kolfe Keranio subcity, Addis Ababa, during the 2024/25 academic year. It comprised students from schools implementing the SFP (intervention group) and comparable public primary schools without school feeding (comparison group). Students who were officially enrolled and attended school for at least one semester were included, while those with incomplete records or who transferred during the study period were excluded.

### 2.4. Inclusion and Exclusion Criteria

#### 2.4.1. Inclusion Criteria

Students who were enrolled in selected public primary schools in Kolfe Keranio subcity, Addis Ababa, during the 2024/25 academic year were included in the study. Eligibility required that students had attended schools either implementing an SFP or comparable schools without the program and had been enrolled for at least one academic semester during the study period, with complete and accessible school records necessary for assessing dropout status.

#### 2.4.2. Exclusion Criteria

Students were excluded from the study if they had incomplete or missing school records, transferred into or out of the selected schools during the study period, were absent for a prolonged period that prevented confirmation of enrollment status, or were newly enrolled and had not completed at least one academic semester at the time of data collection.

### 2.5. Sample Size and Sampling Technique

#### 2.5.1. Sample Size

A large sample size was recommended to preserve the quality of the data. Since they represent the remaining primary schools in the Kolfe Keranio subcity, the researcher included all 24 primary schools in Kolfe Keranio that started instruction in 2009 E.C. By obtaining information from student rosters, the dropout rates were analyzed by contrasting students who attended each school between 2009 and 2011 E.C. with those who attended after the SFP was implemented between 2012 and 2015 E.C.

#### 2.5.2. Sampling Techniques

In this investigation, a purposive multistage sampling strategy was used. Kolfe Keranio subcity was intentionally selected as the study area because it is one of the Addis Ababa subcities implementing the SFP and maintains relatively complete and accessible school administrative records. The earlier reference to “eleven subcities” has been corrected, as Kolfe Keranio is a single administrative subcity.

The second stage involved the purposeful selection of public primary schools in Kolfe Keranio subcity based on predetermined criteria, such as the length of time the schools participated in the SFP, the consistency with which the program was implemented, and the completeness of the school records concerning enrollment, dropout, and academic performance. The study comprised schools that had used the SFP long enough to compare results before and after the intervention and that kept accurate administrative records.

In order to ascertain student dropout rates both before and after the implementation of the SFP, school roster data were examined in the final stage, which covered all eligible grades within the chosen schools. Instead of sampling specific pupils, administrative data at the school level as a whole was examined.

The purposive strategy was appropriate given the study’s goal of assessing program‐associated changes using trustworthy administrative data, even though random sampling was not used. However, the results may not be as broadly applicable to other schools with comparable program implementation features due to this sampling technique. Therefore, the findings should be taken as context‐specific evidence that is pertinent to urban public primary schools that are launching school food programs in similar circumstances.

### 2.6. Data Source

To learn more about how SFP affects student dropouts in the specified location, the study uses secondary data sources. The student roster card served as the source of the secondary data. The researcher collected data from 24 chosen schools between 2009 and 2015 E.C. using student roster cards. Since all elementary schools receive SFP, the lack of experimental and control groups made the data collection technique necessary. Using observational methods, the researcher collected data from all primary school [1–8] rosters for the years 2009–2015 E.C.

### 2.7. Variables

#### 2.7.1. Dependent Variables

School dropout rate (status of dropping before and after the program).

#### 2.7.2. Independent Variables

In‐SFP.

### 2.8. Variable Measurement and Instrument

#### 2.8.1. Instrumentation

Secondary quantitative data sources were used in the investigation. Each academic year’s change in enrollment and dropout rate was measured using the student roster card. The record offices of the chosen school are the source of the school record. For comparison, the same students’ records from every school year were gathered.

### 2.9. Operational Definition

 Absenteeism is a measure that indicates the number of days a primary school student missed attending in a specific academic period. However, for analytical purposes, absenteeism is calculated as the attendance rate [20].
 School feeding might be essentially characterized as the availability and provision of sufficient food for kids in terms of quantity, quality, safety, and sociocultural acceptability. Meals served on school property are typically referred to as school feeding; however, there are other feeding options, such as take‐home rations (THR). THR are given to the children’s families, typically on the condition that their children attend school, but the idea of in‐school meals suggests that food is given to students while they are in class.
 Standard definition of SFP: An SFP is defined as a structured social protection and education intervention that provides regular meals or snacks to children while they are attending school, with the primary objectives of improving school enrollment, attendance, retention, and learning outcomes, while also contributing to child nutrition and food security. SFPs may be implemented as in‐school meals or THR, and they are widely used as a strategy to reduce short‐term hunger, enhance cognitive performance, and mitigate socioeconomic barriers to education among vulnerable populations [21].
 The SFP is an educational intervention that aims to increase school participation and learning achievements by providing free meals to school children [20].
 School input supply is the provision of educational input supplies such as exercise books, pencils and papers, and school bags, to increase access and participation for unprivileged students [20].
 Dropout rate is the percentage of children enrolled in a given year who did not finish the school year. The dropout rate is thus the ratio of children who did not complete the school year to the number of children enrolled in school that year [22].
 Enrollment: This figure is the official figure recorded at the beginning of the school year. There is usually an enrollment period at the beginning of the school year. After the enrollment period has closed, children may still enroll or leave [22]. The official figure for the year nevertheless remains the same as recorded at the end of the enrollment period.
 Food security: The commonly accepted definition of food security is the one that is defined by FAO: “Food security exists when all people, at all times, have physical, social and economic access to sufficient, safe and nutritious food, which meets their dietary needs and food preferences for an active and healthy life” [23].
 Preprimary and primary school: Preprimary is a school that is before grade 1, and primary is a school from grade 1 to 8. This level of education is divided into the first cycle (grades 1–4) and the second cycle (grades 5–8) education.

### 2.10. Reliability and Validity

By gathering similar data from preimplantation and postimplementation roster records, the study ensured its data were reliable. To ascertain whether the two groups’ average dropout rates showed comparable trends, the researcher assessed the parallel trend assumption. By using this method, the researcher was able to reduce the possibility of skewed data when comparing the two groups. Additionally, the risk of attrition bias was reduced because the pre‐ and postgroups were selected at the same follow‐up intervals and referred to the baseline from a year earlier by looking at their roster. A typical sample size (first and second cycle primary) was also used in the study. As such, it has a high level of external validity.

### 2.11. Data Collection

The study focused on public primary schools that began implementing the SFP in 2009 E.C. to ensure a uniform intervention start time and allow for a valid comparison of dropout rates before and after program implementation. Schools that started the program earlier or later were excluded to minimize variability related to differing exposure durations. Student rosters obtained from school administrative records were used to calculate annual dropout rates. A dropout was defined as a student who officially enrolled at the beginning of the academic year but did not complete the year and was not recorded as transferred. For the pre‐ and postintervention comparison, consistent academic years were selected, and only students continuously enrolled at the start of each academic year were included; students who transferred in or out during the academic year were excluded from dropout calculations to avoid misclassification. All 24 eligible public primary schools in Kolfe Keranio subcity were included in the study, constituting a census of schools meeting the inclusion criteria. Including all eligible schools enhanced data completeness, reduced selection bias, and ensured adequate representativeness of the study setting, thereby strengthening the validity and reliability of the findings.

### 2.12. Data Analysis

This study employed a quantitative quasi‐experimental design using a one‐group pretest–post‐test approach to assess the effect of the SFP on student dropout rates in Kolfe Keranio Public Primary School. The SFP was treated as the independent variable, defined as the implementation of regular school meals, while the school‐level dropout rate was the primary dependent variable.

The dropout rate was operationalized as the proportion of students who discontinued schooling during an academic year relative to the total number of enrolled students, as recorded in official school administrative registers. Preintervention data were obtained from the academic year preceding the introduction of the SFP, while postintervention data were extracted from the academic year following sustained program implementation. These clearly defined time intervals allowed for a temporal comparison of dropout rates before and after the intervention.

School administrative records were reviewed to obtain pre‐ and postintervention dropout data. Descriptive statistics, including means and percentages, were used to summarize trends in dropout rates across the two time periods. The findings were presented using tables and graphical displays to facilitate comparison.

To determine whether there was a statistically significant change in dropout rates following the implementation of the SFP, a paired‐samples *t*‐test was applied. This test was selected because it is appropriate for comparing mean differences between two related measurements taken from the same school population at different time points. Before conducting the analysis, key statistical assumptions for the paired‐samples *t*‐test were assessed, including the normality of difference scores and the presence of outliers, to ensure the validity of the results.

All statistical analyses were conducted using the Statistical Package for the Social Sciences (SPSS), version 27, and statistical significance was determined at a *p*‐value of less than 0.05. Potential confounding factors such as changes in school administration, concurrent educational interventions, and broader socioeconomic conditions were considered in the interpretation of the findings. However, due to the absence of a control group resulting from the universal implementation of the SFP in Addis Ababa public schools, the results are interpreted as program‐associated changes rather than definitive causal effects.

### 2.13. Ethical Consideration

Ethical clearance for this study was obtained from the Institutional Review Board (IRB) of the GT Technology College (Reference No. GTTC/25/08/2024). Informed consent was not used because the study was retrospective. But by keeping the information that was extracted private and making sure that it could only be utilized for study‐related objectives, the data’s secrecy was preserved. The participant’s informed consent waiver was authorized by the institutional ethical review board of the GT Technology College in Gondar, Ethiopia. The study was conducted in accordance with the ethical principles outlined in the Declaration of Helsinki (2013 version).

## 3. Results

### 3.1. Eight‐Year Trend of School Enrollments and Dropout Rate Per Grade and Sex (2009–2015 E.C.)

From the school in the Kolfe Keranio subcity, an eight‐year trend was found. A retrospective study methodology was used to incorporate data from 24 elementary schools that started instruction in 2009. For each grade, the year with the highest dropout rate was analyzed as follows: male students were more prevalent in grade one in 2009 (4.69%); female students were more prevalent in grade two in 2010 (5.07%); female students were more prevalent in grade three in 2009 (3.37%); male students were more prevalent in grade four in 2009 (3.02%); male students were more prevalent in grade five in 2010 (3.44%); male students were more prevalent in grade six in 2009 (3.93%); male students were more prevalent in grade seven in 2009 (3.91%); and, finally, the highest dropout rate for grade eight was in 2009 (2.13%), with a higher dropout rate among male students (1.55%). According to the data, the majority of students left school during the 2009 academic year, with male students being especially vulnerable (Table 1).

**TABLE 1:** Eight‐year trend in dropout rate by grade and sex category in Kolfe Keranio subcity (2009–2015 E.C.), Addis Ababa, Ethiopia, 2024.

**Year (EC)**		**2009**			**2010**			**2011**			**2012**			**2013**			**2014**			**2015**		
**G**	**Sex**	**M**	**F**	**T**	**M**	**F**	**T**	**M**	**F**	**T**	**M**	**F**	**T**	**M**	**F**	**T**	**M**	**F**	**T**	**M**	**F**	**T**

G1	E	2401	2545	4946	2271	2407	4678	2358	2499	4857	3420	3546	6966	3937	4109	8046	4297	4129	8426	5608	4798	10,406
	D	115	117	232	82	110	192	56	95	151	39	44	83	106	114	220	74	78	152	119	98	217
	P (%)	4.97	4.60	4.69	3.61	4.57	4.10	2.37	3.80	3.10	1.14	1.24	1.20	2.69	2.77	2.73	1.72	1.89	1.80	2.12	2.04	2.09

G2	E	2192	2340	4532	2073	2213	2486	2153	2298	4451	3321	3489	6810	4132	4066	4598	3232	3342	6574	4051	6125	10,176
	D	58	69	127	54	72	126	46	58	104	27	28	55	63	85	148	28	44	72	90	110	200
	P	2.65	2.95	2.80	2.61	3.25	5.07	2.14	2.52	2.34	0.81	0.80	0.81	1.52	2.09	3.22	0.87	1.32	1.10	2.22	1.80	1.96

G3	E	2088	2397	4485	1975	2267	4242	2051	2354	4405	3170	3375	6545	4181	4257	8438	3212	3213	6425	3071	5281	8352
	D	68	83	151	60	75	135	35	37	72	23	21	44	52	51	103	32	48	80	71	77	148
	P	3.26	3.46	3.37	3.04	3.31	3.18	1.71	1.57	1.63	0.73	0.62	0.67	1.24	1.20	1.22	1.00	1.49	1.25	2.31	1.46	1.77

G4	E	2224	2345	4569	2103	2218	4321	2184	2303	4487	3085	3173	6258	4038	4200	8238	3454	3305	6759	3332	5232	8564
	D	68	70	138	59	56	115	49	51	100	19	31	50	57	69	126	29	26	55	104	58	162
	P	3.06	2.99	3.02	2.81	2.53	2.66	2.24	2.22	2.23	0.62	0.97	0.80	1.42	1.64	1.53	0.84	0.79	0.81	3.12	1.11	1.89

G5	E	2169	2414	4583	2051	2283	4334	2130	2374	4504	3078	3342	6420	3927	3941	7868	3403	3265	6668	4280	4644	8924
	D	80	59	139	81	68	149	61	52	113	25	37	62	80	70	150	38	34	72	98	70	168
	P	3.69	2.44	3.03	3.95	2.98	3.44	2.86	2.19	2.51	0.81	1.11	0.97	2.04	1.78	1.91	1.12	1.04	1.08	2.29	1.51	1.88

G6	E	2047	2469	4516	1936	2235	4171	2010	2321	4331	3059	3219	6278	3782	4225	8007	2854	2932	5786	3082	5519	5901
	D	68	71	139	76	64	140	66	54	120	37	23	60	75	56	131	41	33	74	60	65	125
	P	3.32	2.86	3.93	2.86	3.36	3.28	2.33	2.33	2.77	1.21	0.71	0.96	1.98	1.33	1.64	1.44	1.13	1.28	1.95	1.18	2.12

G7	E	2329	2705	5034	2203	2558	4761	2288	2656	4944	3336	3628	6964	3707	4082	7789	3019	3235	6254	4290	4150	8440
	D	103	98	201	92	90	182	86	87	173	42	31	73	88	91	179	45	26	71	119	58	177
	P	4.42	3.62	3.99	4.18	3.52	3.82	3.76	3.28	3.50	1.26	0.85	1.05	2.37	2.23	2.30	1.49	0.80	1.14	2.77	1.39	2.10

G8	E	2062	2842	3104	2423	3243	5666	2460	2620	5080	2516	3368	5884	3443	3735	7178	1407	1787	3194	2987	4408	7395
	D	32	34	66	29	30	59	4	6	10	21	25	46	72	60	132	32	31	63	67	31	98
	P	1.55	1.20	2.13	1.20	0.95	1.04	0.16	0.23	0.20	0.83	0.74	0.78	2.09	1.61	1.84	2.27	1.73	1.97	2.24	0.70	1.33

*Note:* *Source*: By compiling data from each primary school, each woreda, and the subcity yearly report.

Abbreviations: D = Dropout, E = Enrollment, G = Grade, P = Percentage.

### 3.2. Trend in Number of Students’ Enrollment and Dropout (2009–2015 E.C.)

**FIGURE 1:**
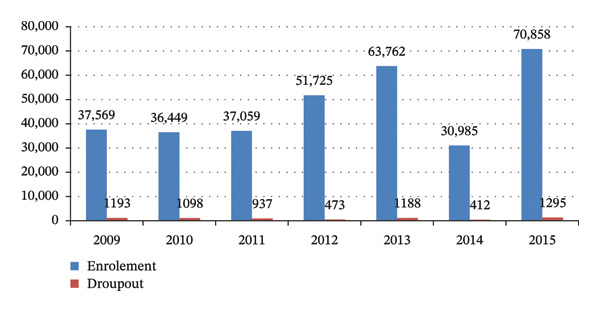
Trend in number of students’ enrollment and dropout (2009–2015 E.C.) in Kolfe Keranio subcity, Addis Ababa, Ethiopia, 2024.

As shown in the figure below, student enrollment and dropout trends differed markedly before and after the implementation of the SFP. During the preimplementation period (2009–2012 E.C.), enrollment levels were relatively lower, and dropout rates were comparatively higher, with the highest number of dropouts recorded in 2009 E.C. (1193 students). Following the introduction of the SFP in 2013 E.C**.**, enrollment showed a consistent upward trend, reaching its highest level in 2015 E.C. (70,858 students)**,** compared with 63,762 students in 2013 E.C**.** Although a temporary increase in dropout was observed in 2015 E.C. (1295 students), the overall postimplementation period was characterized by improved student retention alongside increased enrollment. These trends suggest that the implementation of the SFP was associated with enhanced school participation and may have contributed to improved enrollment stability over time (Figure 1).

From the analysis result, enrollment and dropout were highest in 2015 E.C. and lowest in 2014 E.C., respectively. In 2015, the highest enrollment might be due to community awareness, governmental effort, etc., while in 2014, E.C., the lowest enrollment might be due to the presence of some conflict in the study area.

### 3.3. Trend of Cumulative School Dropout Rate by Sex Category From 2009 to 2015 E.C.

**FIGURE 2:**
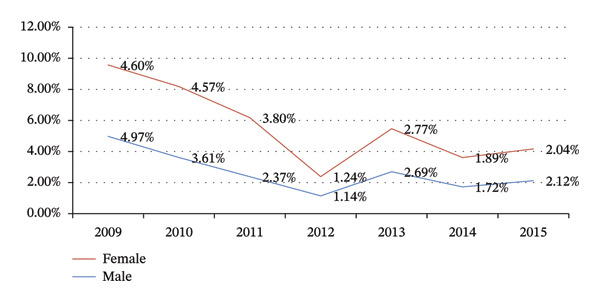
Trend of cumulative school dropout rate by sex category from 2009 to 2015 E.C. in Kolfe Keranio subcity, Addis Ababa, Ethiopia, 2024.

The line graph below reveals that in each year, female students had the highest dropout rate compared to male students before as well as after the implementation of the SFP in Kolfe Keranio subcity (Figure 2).

In Figure 2, the highest dropout rate is observed among female students before as well as after the implementation of the school feeing program. This might be because females were engaged in home activities.

### 3.4. Trend of Cumulative School Dropout Rate From 2009 to 2015 E.C.

**FIGURE 3:**
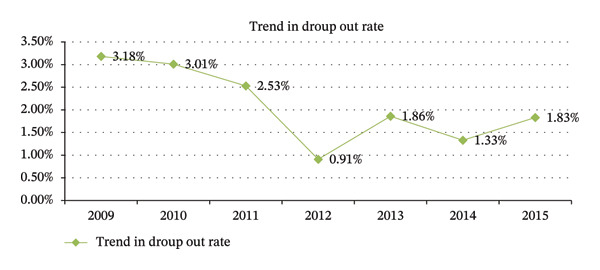
Trend of cumulative school dropout rate from 2009 to 2015 E.C. in Kolfe Keranio subcity, Addis Ababa, Ethiopia, 2024.

This study demonstrated that the dropout rate declined substantially during the preimplementation period of the SFP, decreasing from 3.18% in 2009 E.C. to 0.91% in 2012 E.C. Following the implementation of the SFP in 2013 E.C., dropout rates exhibited a fluctuating pattern between 2012 and 2015 E.C., with periods of both increase and decrease. Nevertheless, the overall magnitude of dropout during the postimplementation period (2013–2015 E.C.) remained consistently lower than in the preimplementation years. This pattern suggests that the introduction of the SFP may have contributed to sustaining reduced dropout rates despite year‐to‐year variations (Figure 3).

The analysis results revealed that even if the dropout rate decreased, the dropout rate before implementation was higher than after the implementation of the SFP. This might be due to the introduction of the SFP implementation, as well as some community and government efforts.

### 3.5. Effect of SFP on Student Dropout Rate

This study examined the effect of the SFP before the implementation of the SFP (2009–2011 E.C.) and after the implementation of the SFP (2012–2015 E.C.). Paired sample *T*‐test was used to identify whether the decreased trend in dropout was really due to SFP implementation or due to other causes such as improving infrastructure, parent education seeking behavior, availability, accessibility, etc.

Table 2 shows the effect of school feeding on the dropout rate. The cumulative average of school feeding beneficiaries before SFP implementation and after SFP implementation was 9.42 and 5.78 hundred, respectively. The mean cumulative score difference due to the SFP was found to be 3.65% (95%CI: 1.68, 5.62) (*t* = −34.38, *p* = 0.003) (Table 2).

**TABLE 2:** Effect of school feeding program on student dropout rate in Kolfe Keranio subcity, Addis Ababa, Ethiopia, 2024.

**Paired samples test**									
		**Paired differences**					***t***	**df**	**Sig. (2-tailed)**
		**Mean**	**Sth. deviation**	**Sth. error mean**	**95% confidence interval of the difference**				
					**Lower**	**Upper**			
Pair 1	Before SFP‐ After SFP	3.64625	2.35658	0.83318	1.67610	5.61640	4.376	7	0.003

To identify the real cause of the decrease in dropout rate, a paired sample *t*‐test was applied. If the *p*‐value is less than 0.05, considered significant, the analysis result indicates that the SFP contributes to a decrease in the dropout rate. From Table 2, it is observed that the dropout rate decreased by 3.65 times after the implementation of the school feeding. From the result, it was concluded that the implementation of the SFP led to a decrease in dropout rate.

## 4. Discussion

In many underdeveloped countries, like Ethiopia, the dropout rate is a serious public health concern. Children may choose not to attend school for a variety of reasons, including social, economic, and other systemic barriers, according to prior studies. As a result, many interventions with various elements, like SFSIS, are essential for sustainably improving enrollment patterns. Thus, during the past 20 years, the primary intervention meant to improve educational achievements in both developed and developing nations has been the development of SFPs [24]. It was hypothesized that SFP is one of the factors influencing the dropout rate, aiming to reduce this rate by offering incentives to children for their daily school attendance [22].

The eight‐year trend analysis of Kolfe Keranio Public Primary School’s SFP’s pre‐ and postimplementation periods was the main topic of this study.

According to the results, grades 1, 3, 4, and 6–8 had notable dropout rates in 2009 E.C., while grades 1 and 5 had higher dropout rates in 2010 E.C. According to the data, female students were more likely to drop out between 2009 and 2015 E.C.

According to the analysis, the cumulative dropout rates were 3.18%, 3.01%, 2.53%, 0.91%, 1.86%, 1.33%, and 1.83% from 2009 to 2015. The data showed variations in dropout rates from 2012 to 2015 E.C., and the trend in dropout rates showed a decrease from 3.18% to 0.91% between 2009 and 2012. According to earlier studies, only 2.5% of primary school‐age children dropped out before the SFP was put into place. However, the findings showed that just 1% of pupils dropped out of school after the school food program was implemented [25]. Additionally, a different study carried out in Bishoftu showed the annual dropout rates for every home, represented as a proportion of the children enrolled in school that year. The numbers are, respectively, 0.002 and 0.009. This indicates that 0.2% of students from schools that took part in the SFP and 0.9% of students from schools that did not take part in the program attended primary school during the research period but left that same year [26].

According to this study, the cumulative average of SFP recipients was 9.42% before and 5.78% after the program was put into place. The school nutrition program was found to be responsible for a mean cumulative score difference of 3.65% (95% CI: 1.68, 5.62) (*t* = −34.38, *p* = 0.003). Consequently, it can be said that the SFP has made a substantial contribution to the beneficiary children’s decreased dropout rate. A study by Tsion A. Desalegn (2021) provides more support for this result, showing that children who participated in the SFP had a considerably lower dropout rate (1.3%) than nonparticipants (5.8%) (*p* = 0.007). Furthermore, compared to their peers, nonbeneficiary youngsters had a sixfold increased chance of dropping out (*p* = 0.008). The idea that SFP in Ethiopia contribute to better educational outcomes was supported by a study by Destaw et al. that found that the termination of SFP in rural Ethiopia increased the dropout rate among girls by 7% when compared to the control group [27].

## 5. Conclusions

This study examined the impact of the SFP on dropout rates in public primary schools in Kolfe Keranio subcity, Addis Ababa. The findings show a measurable reduction in school dropout following program implementation, indicating improved student retention among beneficiaries. The decline in dropout rates between the preintervention and postintervention periods suggests that addressing short‐term hunger through school feeding supports continued school participation in low‐income urban settings.

The results provide empirical evidence that SFPs can improve educational outcomes by reducing food‐related barriers to attendance and persistence. By alleviating hunger during the school day, the program likely enhanced students’ ability to remain enrolled, particularly among children from economically vulnerable households.

However, the findings should be interpreted with caution. The one‐group pretest–post‐test design without a comparison group limits causal attribution, and unmeasured contextual factors may have influenced the results. Despite these limitations, the study adds important school‐level evidence on dropout reduction in urban Ethiopia and offers policy‐relevant insights to strengthen education and social protection strategies.

**Nomenclature**
AAREB: Addis Ababa Regional Education Bureau
CSA: Central Statistics Agency
ESDP: Education Sector Development Program
HGSF: Home‐grown School Feeding
MOE: Ministry of Education
NGO: Nongovernmental Organization
OCL: Observation Check List
SFSIS: School Feeding and School Input Supply
SFP: School Feeding Program
USD: United States Dollar
WFP: World Food Program.

## Author Contributions

Mequanente Dagnaw, Eden Hailu, and Suleyman Mohammed Arage initiated the idea of research, conceptualizing, developing a proposal, doing analysis, and writing up the manuscript. Mequanente Dagnaw performed analysis, reviewed the manuscript, and corrected it.

## Funding

No funding was obtained for this study.

## Disclosure

All the authors have approved the manuscript by preparing it for submission.

## Ethics Statement

Ethical clearance for this study was obtained from the Institutional Review Board (IRB) of the GT Technology College (Reference No. GTTC/25/08/2024). Informed consent was not used because the study was retrospective. But by keeping the information that was extracted private and making sure that it could only be utilized for study‐related objectives, the data’s secrecy was preserved. The participant’s informed consent waiver was authorized by the institutional ethical review board of the GT Technology College in Gondar, Ethiopia. The study was conducted in accordance with the ethical principles outlined in the Declaration of Helsinki (2013 version).

## Consent

The authors have nothing to report.

## Conflicts of Interest

The authors declare no conflicts of interest.

## Data Availability

The corresponding author will have the right access to the data upon request.
